# Effects of Water Deficit and Heat Stress on *Nicotiana langsdorffii* Metabolomic Pattern Modified by Insertion of *rolD* Gene from *Agrobacterium rhizogenes*

**DOI:** 10.3390/metabo10080310

**Published:** 2020-07-29

**Authors:** Elisa Scalabrin, Marta Radaelli, Gabriele Capodaglio

**Affiliations:** Department of Environmental Sciences, Informatics and Statistics, Ca’Foscari University of Venice, Via Torino 155, Mestre, 30173 Venezia, Italy; marta.radaelli@unive.it (M.R.); capoda@unive.it (G.C.)

**Keywords:** abiotic stresses, ionomics, metabolomics, *Nicotiana langsdorffii*, plant metabolome, *rolD*

## Abstract

Abiotic stresses are major factors that negatively affect plant growth and productivity. Plants have developed complex strategies to ensure their survival and reproduction under adverse conditions, activating mechanisms that involve changes at different metabolic levels. In order to select stress-resistant species, research has focused on molecular studies and genetic engineering, showing promising results. In this work, the insertion of the *rolD* gene from *Agrobacterium rhizogenes* into *Nicotiana langsdorffii* plants is investigated, in order to assess the potential of this genetic modification towards mitigating water and heat stresses. Different approaches were combined: a high-throughput metabolomics and ionomics study was performed, together with the determination of important plant phytohormones. The aim was to identify the influence of abiotic stresses on plants and to highlight the effects of the *rolD* genetic modification on plant stress response. The most relevant compounds for each kind of stress were identified, belonging mainly to the classes of lipids, acyl sugars, glycosides, and amino acid derivatives. Water stress (WS) determined a decrease of elements and secondary metabolites, while amino acids and their derivatives increased, proving to be key molecules in this type of stress. RolD plants exposed to high temperature stress (HS) presented higher dry weight levels than controls, as well as increased amounts of K and adenosine and lower levels of damage-associated metabolites, suggesting the increased resistance of *rolD*-modified plants toward HS.

## 1. Introduction

Among the major environmental-adverse conditions that land-use is expected to face under climate change pressure, drought and heat stress represent those that are the most expectable, widespread, and have the highest impact on human activities [1]. Yield safety should, therefore, be improved on the basis of new knowledges about plant development and its responses to stress [2]. Plants are able to deal with highly variable environmental stresses by activating a complex system of responses. However, abiotic stresses can highly compromise plant growth and survival; heat stress conditions, in particular, are known to alter growth and photosynthesis, damage cell membranes, increase transpiration [3], and modify hormonal homeostasis [2]. Changes in the lipidic composition of cellular membranes are often observed [4]. Water deficiency influences the main physiological processes of plants, reducing photosynthesis and mineral nutrient uptake; alterations of respiration rate and of membrane bilayers have also been reported [5,6]. Abiotic stresses highly affect primary and secondary plant metabolism, influencing mineral nutrient levels and the production of metabolites. For this reason, the effect of abiotic stresses can be assessed by monitoring the variation in levels of different phytoregulators. Plant responses to abiotic stresses include alterations in the levels of secondary metabolites, which play a crucial role in adapting to changing environments [1]. Among many others, phenolics provide protection against reactive oxygen species (ROS), which are produced in the primary response to both heat and water stresses [7]. Compatible osmolytes, such as amino acids, sugars, organic acids, and amines [8,9], are produced to balance the plant’s osmosis. Some inorganic ions, moreover, such as K, Ca, and Na, are essential in enzymatic processes and in the maintenance of cellular turgidity and electroneutrality [10].

In recent decades, research studying the molecular mechanisms of stress responses has gained much more interest and, in parallel, genetic modifications relating to stress tolerance have also shown promising results, which may ultimately be applied to agriculturally and ecologically important plants [11]. Among the genetic modifications studied, the integration of the rat glucocorticoid receptor (GR receptor) has been shown to modify the hormonal pattern of *Nicotiana* plants [12], leading to higher resistance to nematode infections [13] and against water and chemical stresses in *Nicotiana langsdorffii* [14,15]. The *rol* genes from *Agrobacterium rhizogenes*, when naturally engineered in plants, have been shown to induce multiple physiological and biochemical alterations in transformed plants [16,17], activating secondary metabolic processes and enhancing the plant’s response to abiotic and biotic stresses [16,17,18,19]. The *Nicotiana* genus (family of *Solanaceae*) has been extensively investigated in genetic and physiological studies; in particular, the integration of *rolC* and GR genes in *N. langsdorffii* and *N. glauca* has shown interesting results for the improvement of plant resistance to different abiotic stresses [10,12,14,15,18,20,21,22,23]. Among the *rol* genes from *A. rhizogenes*, *rolD* has been investigated to a lesser extent. However, its integration in tobacco plants has demonstrated the inducement of various metabolic effects. Particularly, *rolD* insertion has been shown to determine the accumulation of proline [24] by encoding ornithine cyclodeaminase, an enzyme catalyzing the conversion of ornithine to proline. Proline is a well-known bio-marker for water deficit [25], playing an important role as an osmolyte, an ROS scavenger, and a molecular chaperone stabilizing the structure of proteins, thereby protecting cells from damages caused by stress [26,27]. Moreover, *rolD* gene insertion has been shown to determine an imbalance of the hormonal status of plants, with higher level of cytokinins and lower levels of auxins [16], which could ultimately induce the formation of sun-type chloroplasts, which are more tolerant against water and temperature stresses [5]. For these reasons, in this study, we investigated the potential of *rolD* insertion in *Nicotiana langsdorffii* in resistance against water and heat stress. In order to achieve a highly comprehensive profile of the major metabolic changes associated with genetic modification and exposure to stress, various analytical approaches were employed. As plant metabolites are the results of cellular functions, their levels may reveal the physiological state of an organism. Particularly, the balance between defense, signaling, and damage metabolites can be useful to assess plant tolerance to abiotic stresses [7]. An untargeted metabolomics analysis was performed, which permitted us to simultaneously analyze the changes of a high number of compounds and highlight the roles of previously unknown molecules involved in stress response. Moreover, selected phytohormones—salicylic acid (SA) and jasmonic acid (JA)—which have been shown to be important biomarkers in the abiotic stress response of *N. langsdorffii* [14], were quantitatively determined. Finally, the ionomics profile was traced, in order to obtain additional information about the plant health status and identify the mechanisms underlying the plant stress physiological response.

This work presents the results of a high-throughput metabolomics and ionomics study of *rolD* transgenic *N. langsdorffii* plants exposed to water stress (WS) and high temperature stress (HS). Wild-type plants and *GR* and *rolC* genetically modified *N. langsdorffii* plants have already been considered in previous studies [14,15,23]. Our aim was to identify the influence of abiotic stresses on plants and to highlight the effects of *rolD* genetic modification on plant stress response. Different approaches were combined, proving to be effective in the unraveling of the rerouting of the plant metabolome during stress, in order to highlight the possible higher resistance of *rolD* plants towards heat stress.

## 2. Results and Discussion

### 2.1. Dry Weight and Chemical Characterization

Non-stressed transgenic plants (RolD) did not show significant growth differences, with respect to fresh weight, in comparison to transgenic plants exposed to water stress (RolDWS) and to transgenic plants exposed to heat stress (RolDHS); however, the dry weight of RolDHS was significantly higher than that of RolD controls (Table 1).

**Figure 1:**
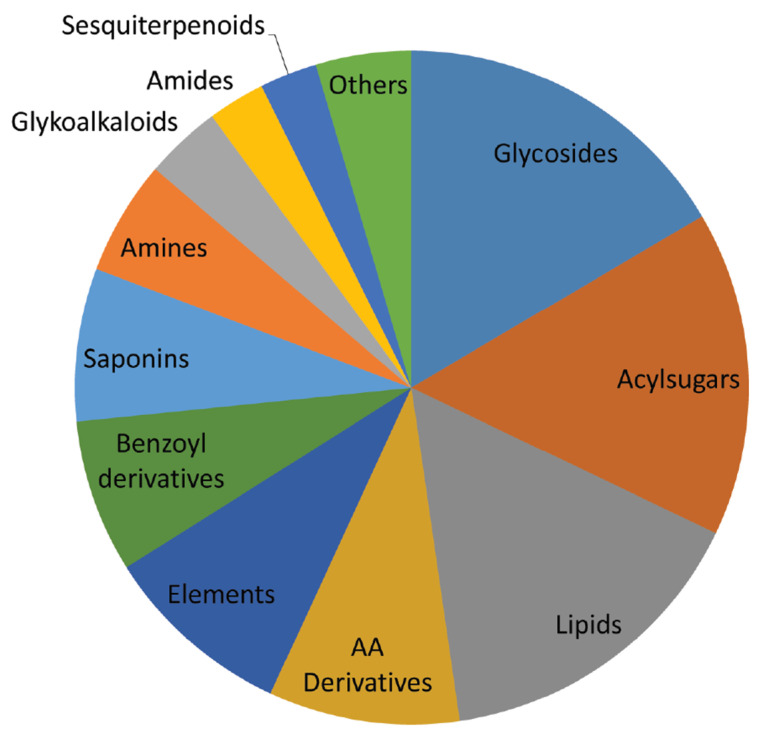
Pie chart of the classes of metabolites identified in *rolD* samples. The category “Others” includes lignans, phenylpropanoids, phytohormones, and nucleosides.

The chemical analysis included the quantitative determination of two important plant phytohormones (SA and JA) and 19 elements (Na, Mg, Al, K, Ca, V, Cr, Mn, Fe, Co, Cu, Zn, Ga, Rb, Sr, Ag, Ba, Pb, and Bi). The untargeted metabolomics analysis permitted the identification of 114 plant metabolites, for an overall analysis of 135 compounds. Of these, only 110 compounds (two phytohormones, 10 elements, and 98 metabolites) had statistically significant variation between the unstressed and HS/WS-stressed plants, of which 18 were glycosidic compounds, 17 were acylsugars, 17 belonged to the class of lipids, 10 were amino acid derivatives, and 10 were elements (Figure 1).

**Figure 2:**
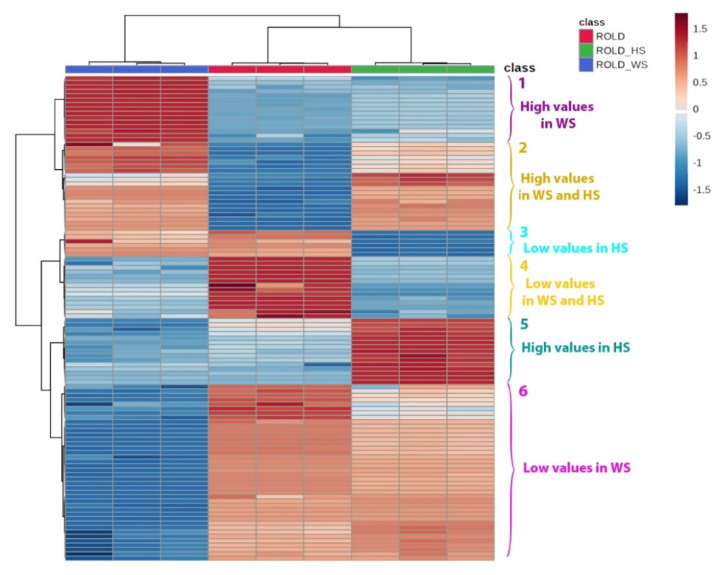
Heatmap of the main metabolites identified in *Nicotiana langsdorffii* RolD (red), RolDWS (blue), and RolDHS (green). The clusters indicate metabolites that showed different patterns in the three classes of samples. Cluster 1: compounds with high values in water stress (WS); Cluster 2: compounds with high values in WS and high temperature stress (HS); Cluster 3: compounds with low values in HS; Cluster 4: compounds with low values in HS and WS; Cluster 5: compounds with high values in HS; and Cluster 6: compounds with low values in WS.

These compounds (see Table S1, Supplementary Files) were statistically elaborated through the construction of a heatmap (Figure 2), which permitted the identification of six clusters of compounds characterized by different variations in the three types of samples, particularly, compounds with high values in WS (cluster 1), compounds with high values in WS and HS (cluster 2), compounds with low values in HS (cluster 3), compounds with low values in HS and WS (cluster 4), compounds with high values in HS (cluster 5), and compounds with low values in WS (cluster 6). The identification of these groups and of the relationships among different compounds permitted us to trace a complete profile of the evolution of the metabolic pattern of transgenic plants exposed to WS and HS and to identify correlations between different metabolites. However, it is noteworthy to highlight that the insertion of the *rolD* gene in *Nicotiana langsdorffii* may have led to changes in plant phenotype, in comparison to wild-type plants, which have been already discussed in previous papers. T-DNA insertion may have influenced the expression of other plant genes, through a mechanism called gene knockdown, and determined alterations in some metabolic pathways and in the levels of the metabolites derived from them. Therefore, the metabolic effects observed in this study may be partly due to the influence of the *rolD* gene on the plant genome, as compared to the expression of the *rolD* gene itself. Nevertheless, the main focus of this work was on the effects of stress exposition on transgenic plants, in comparison to unstressed samples.

### 2.2. Determination of Elements

Nineteen elements (Na, Mg, Al, K, Ca, V, Cr, Mn, Fe, Co, Cu, Zn, Ga, Rb, Sr, Ag, Ba, Pb, and Bi) were determined in all samples, of which only 10 had a statistically significant variation in stressed samples, in comparison to controls. Particularly, V, Rb, and Co were decreased in WS and HS samples; Na, K, Ca, Mn, Fe, and Ag were decreased only in WS samples; and K was increased in HS samples. The interpretation of these data is provided, together with the levels of metabolites and phytohormones, in the following paragraphs, in order to obtain a comprehensive overview of the metabolic changes associated with plant exposure to WS and HS.

### 2.3. Metabolites That Vary in Both WS and HS (Clusters 2 and 4)

**Figure 3:**
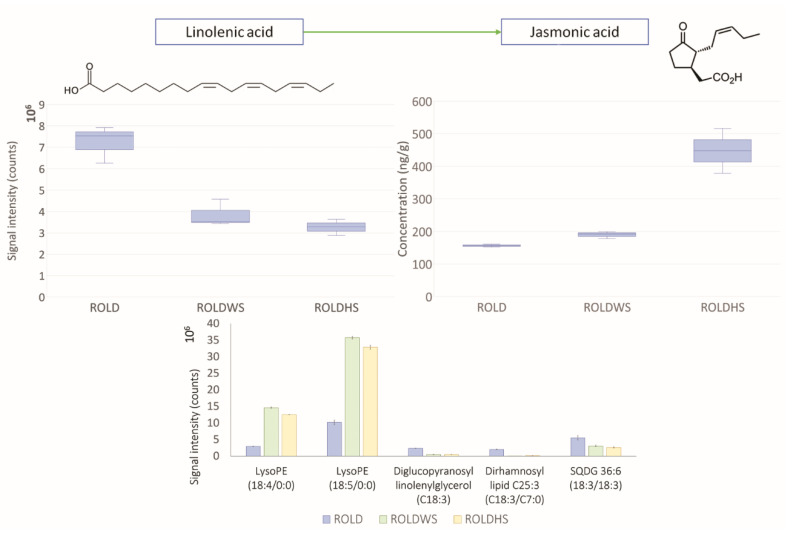
Levels of linolenic acid, jasmonic acid, and the main lipids which varied in both WS and HS samples. Error bars indicate standard deviation and 10^X^ specifies the unit of visualization.

As expected, SA and JA intensities increased in both WS- and HS-stressed samples, confirming the important roles of these two compounds against abiotic stresses [28]. This result clearly indicates the activation of the defense system in the *rolD* plants as a consequence of stress exposure. In our previous study [14], SA and JA showed significant variations only in HS plants of different genotypes (modified for the rat glucocorticoid receptor gene and the *rolC* gene from *Agrobacterium rhizogenes*). This could indicate that, for the *rolD* gene, different mechanisms are involved in the plant response to WS. Some studies have already highlighted that the SA pathway contributes to acquired thermotolerance [29,30] and that SA supplementation to plants exposed to drought enhances the photosynthesis rate and decreases oxidative stress [31]. JA is known to be implicated in drought and heat avoidance, by promoting stomatal closure and modulating root conductivity [32], and has been shown to be essential for thermotolerance in *Arabidopsis thaliana* [33]. In relation to JA enhancement, a decrease of linolenic acid was registered during both stresses; this compound is the main precursor of JA and unsaturated fatty acids. The decrease of linolenic acid during abiotic stresses could represent a consequence of the increased biosynthesis of JA and oxylipins, which are known to be important stress-signaling molecules (Figure 3) [34]. Moreover, the decrease of linolenic and other trienoic fatty acids, especially in chloroplast membranes, has been shown to strongly enhance high-temperature tolerance in plants [35,36].

Lipids, being the main constituents of cell membranes, are one of the main classes of metabolites involved in plant stress responses, especially with respect to high temperature. In this study, we observed an increase in the levels of two lysolipids (18:4/18:5) during both WS and HS, probably as an effect of stress application. Lysolipids have, indeed, been considered as sensitive indicators of stress responses in plants [15,37]; in particular, lysophosphatidylethanolamines (LPEs) have been suggested to be catabolites of hormone-activated phospholipase 2 (PLA2) and to serve as secondary messengers in plants to modulate phospholipase D (PLD) [38,39]. Generally, high temperature stress determines an increase of saturation level of lipids, as a response of the plant to maintain the appropriate membrane fluidity. In our case, however, we observed the increase of content of lipids with a higher unsaturation level (Figure 3). This trend in lipid composition has been already observed in *Festuca arundinacea Schreb*. [40] during HS and in wheat leaf during WS [41].

Both abiotic stresses suppressed other lipids belonging to different classes, such as sulfoquinovosyl (SQDG) and dirhamnosyl lipids, all containing at least a C 18:3 chain. This result suggests that the mechanisms of plant response toward HS and WS were strictly linked to linolenic acid biosynthesis/metabolism, probably in relation to oxylipins and JA production.

**Figure 4:**
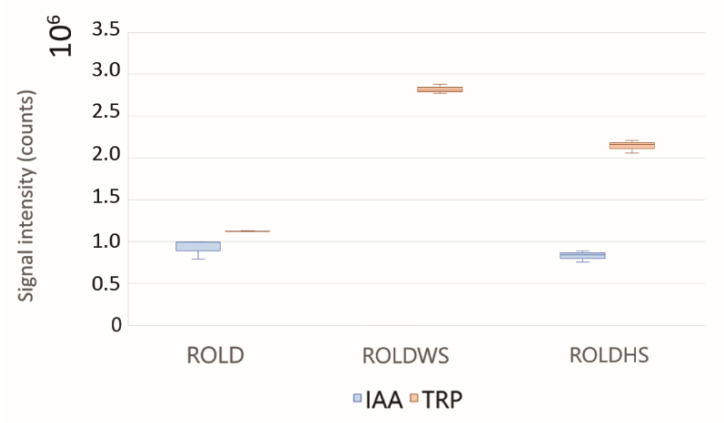
Box plots of tryptophan (TRP) and indole-3-acetic acid (IAA), the most important auxin in plants. 10^X^ specifies the unit of visualization.

It is well-known that drought and heat tolerance strategies can determine a metabolic adjustment, defined as the accumulation of compatible solutes and protective proteins [6]. In particular, increased content of osmolytes, such as amino acids, derived from reduced protein synthesis or general protein breakdown during osmotic stress, are expected [42]. In our case, an increase in the level of tryptophan (TRP) was observed in both HS and WS plants; this compound is one of the least abundant and biosynthetically energy intensive amino acids, which is the precursor of many important metabolites involved in plant defense (e.g., auxin, phytoalexins, and alkaloids), thus playing a critical role in regulating plant development and its response to biotic and abiotic stresses [43]. The high level of TRP may be a consequence of the reduced biosynthesis of auxin under WS and HS (with decreases of 99% and 16%, respectively; Figure 4), as previously reported during abiotic stresses [44,45]. Although proline has been studied most frequently, increases of valine (VAL), isoleucine (ILE), leucine (LEU), phenylalanine (PHE), and methionine have been reported during drought stress [42,46]. In our case, increased levels of the dipeptide valyl valine and homomethionine quinate were detected, indicating the interference of heat and water stresses in the polypeptide pathway [47]. The decrease of homomethionine has been previously reported by Moradi et al. [48] in *Thymus vulgaris*, which is sensitive to water stress, in comparison to *Thymus serpyllum*, which is tolerant to drought stress, suggesting the involvement of this compound in the plant response to abiotic stresses.

During HS and WS, we also observed increases of dicaffeoylspermidine and bis-dihydrocaffeoylspermine, which belong to the group of hydroxycinnamic acid amides (HCAAs); these metabolites have been found to accumulate during various kind of stresses, both abiotic and biotic [49,50], but their mode of action during stress is still not well understood. A common feature of all types of abiotic stresses is oxidative stress induced by the production of reactive oxygen species (ROS). Therefore, the activation of ROS detoxification mechanisms is crucial for the success of drought and heat tolerance strategies [6]. It is well-known that HCAAs have a high antioxidant potential [1] and it has been suggested that they are related to the promotion of the activity of antioxidant enzymes [51], that they are able to limit lipid peroxidation [52], and that they can affect protein kinase and/or phosphatase activities to regulate ion channel functions during stress [49,53]. Moreover, conjugation of polyamines with phenolic compounds significantly reduces their polarity and hydrophilicity, which may favor their translocation and stability. While conjugation can be a means to regulate the pools of both parent compounds and to store phenolics and bioactive polyamines, conjugates are often regarded as final and accumulated products [51].

We also observed the increase of some acylsugar compounds during both heat and water stresses. Acylsugars are metabolites that constitute a significant proportion of leaf biomass in some *Solanaceous* species, such as *Nicotiana*. They are mainly produced and accumulated in the plant’s trichomes and are known to be involved in the defense against pathogens [54,55]. In RolDHS and RolDWS plants, the levels of four acylsucroses were increased: S3:21 (where 3 is the number of acyl groups and 21 the number of carbons), S4:23, S4:24, and S4:26; while the tetraacylsucrose S4:15 was reduced. These results, in agreement with our previous study [15], confirmed the suggestion that the biosynthesis of acylsugars is induced during abiotic stresses. Although the defensive roles of these compounds during heating or drought have not yet been well investigated, our results highlight their possible function in plant responses to abiotic stresses. Moreover, the increase of acylsugars with a high number of carbons (C23–C26) and the decrease of S4:15, with lower molecular weight, indicated that the presence of a long carbon chain was crucial for the defense mechanism against HS and WS.

Another group of compounds that were enhanced in both HS and WS were diosgenin and its glycoside derivatives; these compounds are steroidal saponins, known to be present in the *Solanaceae* genus, which are involved in the biosynthesis of steroidal hormones [56,57]. Previous studies have indicated increases of diosgenin and related steroidal saponins after exposition to water stress in *Tribulus* species [58]. Furthermore, exposure to Cu metal stress has been shown to enhance the production of diosgenin in *Dioscorea bulbifera* L. cultures [59].

**Figure 5:**
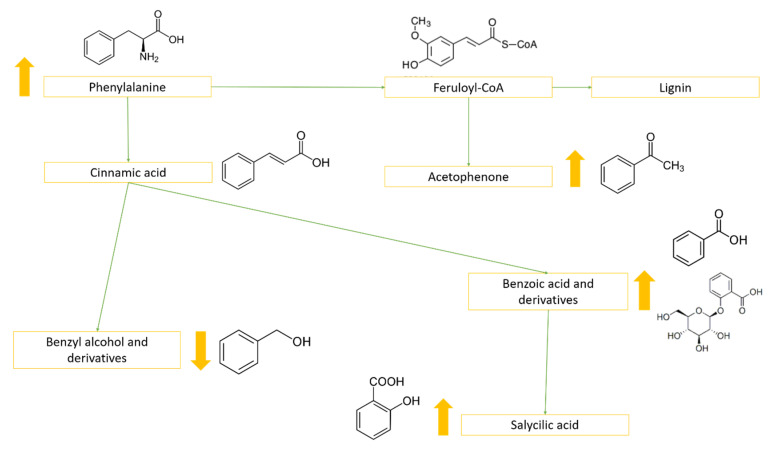
Main biosynthetic pathway steps highlighting the relationships among phenylalanine (PHE), the phenylpropanoid pathway, acetophenone, benzoic acid and its glycosides, and salicylic acid (SA). Yellow arrows indicate the variations during WS exposition.

Both WS and HS clearly induced alterations in the phenylpropanoid pathway and in the benzoic acid biosynthetic route; particularly, changes in the levels of acetophenone and benzoic acid glycosides were observed. These compounds are known to be involved in the defense mechanisms of plants [60,61], probably as an antioxidant response to increased levels of ROS. Moreover, benzoic acid glucoside has been identified as a precursor of SA in tobacco plants and cell cultures [62]. In *Nicotiana tabacum*, indeed, acetophenone glycosides have been suggested to derive from feruloyl-CoA [63], while benzoic acid and its derivatives are known to be produced starting from cinnamic acid (Figure 5). In this context, we observed the increase of phenylalanine (PHE), which is the precursor of both benzoic acid and acetophenone glycosides [64].

Regarding the elemental content, application of WS and HS determined a decrease in Vanadium (V) concentrations, which could be due to a reduction in the intake of this element. However, V is probably involved in the regulation of photosynthesis, as a substitute of an essential cofactor for one of the enzymes of chlorophyll biosynthesis [65]; therefore, its diminution could be related to an increase in its utilization during stress conditions that affect the photosynthesis rate [66].

### 2.4. Metabolites That Vary Only in WS (Clusters 1 and 6)

**Figure 6:**
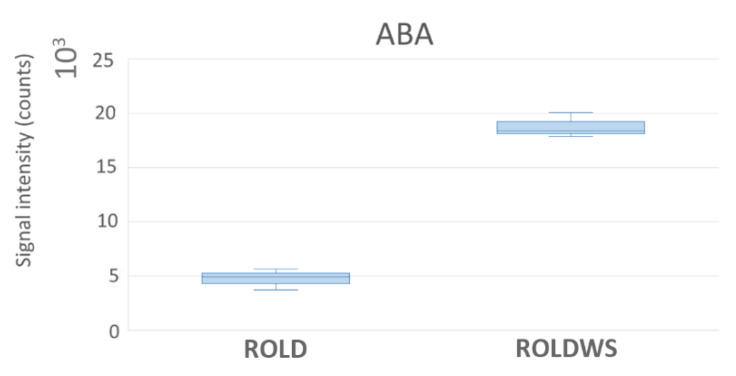
Abscisic acid (ABA) levels in RolD and RolDWS samples. 10^X^ specifies the unit of visualization.

In WS samples, a significant change in abscisic acid (ABA) levels was observed; this compound is considered to be the most important biochemical marker of drought stress, regulating stomatal closure and, therefore, being involved in plant drought avoidance strategies [6]. In our samples, ABA increased up to four-fold (Figure 6), clearly demonstrating the presence of drought stress.

**Figure 7:**
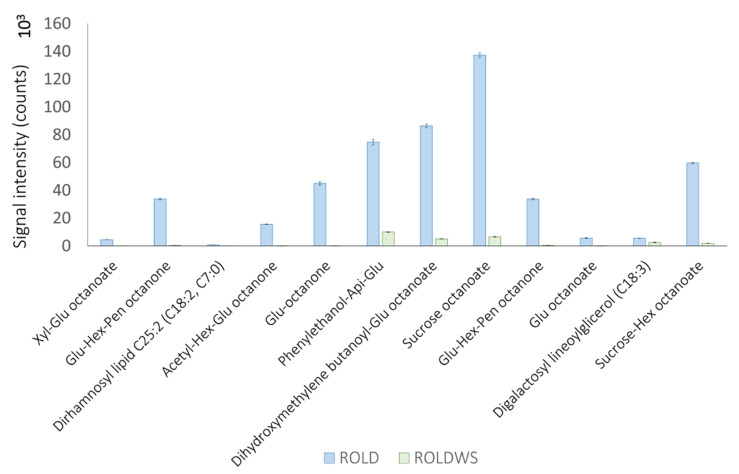
Glycolipids varying in RolDWS samples. Error bars indicate standard deviation and 10^X^ specifies the unit of visualization.

The major part of compounds that showed a statistically significant variation in WS samples tended to decrease (Figure 2, cluster 6), as already highlighted by Scalabrin et al. [15]; particularly, we detected the reduction of acylsugars S4:17, S4:18, S4:19, S4:22, and S3:12; while triacylsucrose S3:24 was enhanced. This result confirms the trend previously observed for HS samples, indicating that the variation of these compounds during stress is highly dependent on the number of carbon atoms of the acyl chains. To the best of our knowledge, this kind of variation has not yet been reported. Moreover, the diminution of many glycosides was observed, probably in the context of metabolic adjustment to establish a drought tolerance strategy [6]. The major part of these compounds were glycolipids or acyl-conjugated; interestingly, nine of which were conjugated with octanoic acid. This result suggests a downregulation of the biosynthesis of glycosides and/or acylsugars, possibly in order to release monomers useful in other metabolic pathways against WS (Figure 7). The role of octanoic acid should be further investigated, in order to understand whether this fatty acid has a specific role during abiotic stresses.

Other lipids that showed significant variations in their levels during WS were lysophosphatidic acids (LPA) and lysophosphatidylglycerols (LPG). Phosphatidic acid is one of the key intermediates of phospholipid biosynthesis and turnover, and is known to be involved in many types of biotic and abiotic stress [67]. PG is the main phospholipid in the photosynthetic membranes in plants and its diminution has already been observed during water stress, partly due to a shift in synthesis or allocation of metabolites to sustain growth [68,69]. Changes in the levels of these compounds clearly indicated the involvement of the photosynthetic system in WS response. Interestingly, in our previous study [11] concerning *rolC* and GR gene insertion in *Nicotiana langsdorffii*, WS did not determine any variations in the intensities of these compounds. Therefore, *rolD* genetic insertion likely differently regulates plant response to WS. However, it is important to note that the observed changes in the metabolome may be ascribed also to the knockdown gene mechanism, caused by T-DNA insertion, more than to *rolD* gene expression. The diminution of these lysolipids could indicate their transformation and utilization as precursors for other molecules (e.g., oxylipins or structural lipids such as monogalactosyldiacylglycerols), or indicate damage of the photosynthetic system [70].

**Figure 8:**
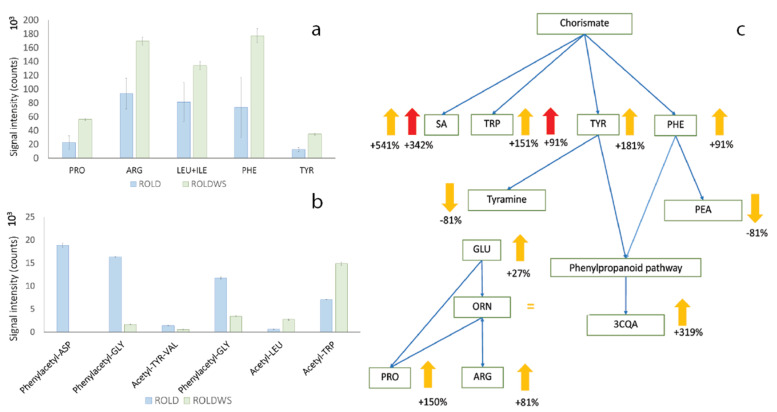
Levels of the main amino acids (**a**) and amino acid derivatives (**b**) in RolD and RolDWS plants; error bars indicate standard deviations and 10^X^ specifies the unit of visualization. Scheme of the biosynthesis (**c**) of the main amino acids and metabolites involved, as derived from the shikimate pathway, and their regulation during WS (yellow arrow) and HS (red arrow). The percentages of changes are shown under the arrows, in comparison to controls.

Among the compounds that showed increased levels, the main group was represented by amino acids; particularly PRO, Arginine (ARG), LEU/ILE, PHE, Tyrosine (TYR), Acetyl-TRP, and Acetyl-LEU (Figure 8a,b), while some other amino acid derivatives (mainly acetyl-phenyl compounds) decreased. PRO is a well-known osmolyte and it has been demonstrated that its elevated levels induce tolerance to water and salt stresses in tobacco and petunia plants [26,27]. Increases in the levels of ARG, ILE, LEU, and PHE have already been reported during drought stress in *Glycine max* [71] and in *Brassica napus* [47]. Increases in the levels of TYR and PHE probably indicate the activation of the phenylpropanoid pathway, as these two amino acids represent the main substrate for the biosynthesis of hydroxycinnamic acids. It must be emphasized that an increase of 3-caffeoylquinic acid (3CQA) was observed, confirming the hypothesis of the enhancement of this biosynthetic pathway. The amino acid derivatives that increased or decreased in WS plants could be involved in metabolic adjustment mechanisms, representing intermediates of the biosynthetic or catabolic processes in the metabolic pathways related to amino acids and proteins (Figure 8); in particular, acetyl-amino acids (Acetyl-TRP and Acetyl-LEU) increased, while those with phenyl substituents decreased (Figure 8b). Zeier [72] reported that the acylation reaction of amino acids may be involved in plant resistance to pathogens and pests by the formation of protective plant metabolites or by the modulation of plant hormone activity. It is already well-known that amino acids are able to conjugate with a variety of compounds and functional groups, including many phytohormones, with important defensive roles [73,74,75]. Our results seem to indicate the induction of acyl-amino acids, confirming the hypothesis proposed by Zeier, while other derivatives generally decreased.

Moreover, two substituted amines—acetyl-tyramine glucoside and acetyl-hydroxy-phenethylamine (PEA) glucopyranose, derived, respectively, from TYR and PHE—were shown to decrease in WS samples (Figure 8). Therefore, it is clear that WS, more than HS, determined a complete re-routing of the amino acid pathway, involving also the biosynthetic pathways deriving from them; thus, amino acids can be considered as the central compounds in the response of RolD against WS.

Among amines, caffeoylputrescine was enhanced, in addition to dicaffeoylspermidine and bis-dihydrocaffeoylspermine, as discussed above. In relation to WS, it has been suggested that the conjugation of hydroxycinnamic acids (which are lignin precursors) with amines could be a storage adaptation, in consequence of the decreased lignification rate in response to water stress [76].

Regarding elemental content, we observed the diminution of Na, K, Ca, Mn, Fe, and Ag. This result is in agreement with previous studies regarding *Nicotiana langsdorffii* modified by the insertion of *rolC* and GR and exposed to abiotic stresses [10,21,23]. The diminution of elements in WS plants could indicate a reduced efficiency of nutrient uptake [77]. In particular, WS determined a significant diminution of K, as already reported [78]. It is well-known that K plays an important role in enzyme activation, photosynthesis, stomatal movements, and water-related processes in plants. Particularly, when K is deficient, the stomata cannot function properly and water losses from plant may reach damaging levels [79]. The diminution of K could indicate stressful conditions in the plant [80], showing that *rolD* genetic modification was not convenient for water deficit conditions.

### 2.5. Metabolites That Vary Only in HS (Clusters 3 and 5)

In general, there was a lower metabolite adjustment in HS plants, in comparison to WS plants and in relation to unstressed RolD plants, differing from what was observed in our previous study, where GR and *rolC* genetic modifications were applied [15].

Heat stress application determined the decrease of a lignan-amide, grossamide, and its direct precursor, feruloyltyramine. Feruloyltyramine is a phytoalexin that is produced especially in *Solanaceae* during the first phases of pathogenic infections [81]. The decrease of these compounds could be related to a reduction of the lignification rate as a consequence of heat stress. The inhibition of lignification during heat stress (35–45 °C) has already been proposed, by Han et al. [82], in rice seedlings showing the downregulation of lignification-related proteins.

In addition to the compounds discussed in Section 2.1, HS application affected the levels of a few other lipidic compounds: LPE (18:3), LPE (18:2), and its glycosylated derivative decreased during heat stress, while a glycosidic derivative of LPA increased. The production of lysophospholipids, which are mainly associated with cellular membranes, and the modulation of their unsaturation rate, could represent an induction of the release of polyunsaturated fatty acids to be used for oxylipin production [83] or damage due to HS application. The increase of trihydroxyoctadecanoic acid, however, indicated the presence of oxidation mechanisms, which primarily affect lipids, probably related to the presence of ROS molecules [15,76]. As already discussed above, LPE may represent important signaling compounds during stress; however, their role is unknown at present. Decreases of LPE (18:2) and LPE (18:3) have been previously observed during HS [84].

Increases were observed in two acylsugars (S4:21, S3:19), in agreement with the results discussed above (Section 2.3 and Section 2.4. The increase of a sulfoquinovosyldiacylglucose (SQDG, 35:6), among the main constituents of thylakoid membranes [85], was also observed, indicating the influence of HS on photosynthetic systems.

In contrast to our previous study [15] considering *rolC* and GR genotypes, the *rolD* genotype showed a lower variation in the composition of membrane lipids after exposition to HS, demonstrating the induction or reduction only of a few structural lipids. This result could indicate that *rolD* plants were less affected by this kind of stress, in comparison to the genotypes examined in the prior study. The hypothesized higher resistance of *rolD* toward HS could also explain the higher dry weight observed for these plants (i.e., RolDHS), in comparison to RolD (Table 1). However, it must be considered that the observed biochemical differences between RolD plants and *rolC* and *gr* genotypes could be related not only to the expression of the inserted transgenes, but also to the influence of the genetic modification on the expression of plant genome. Another compound that was shown to be induced during HS was adenosine, a ribonucleoside that plays a central role in the storage and production of energy for the synthesis of the main metabolites of plants [46]. Moreover, adenosine is structurally similar to cytokinins and is probably involved in their biosynthesis [86]. This increase in adenosine could indicate the induction of its biosynthetic pathway and its metabolism for the production of energy or secondary metabolites. Indeed, one of the stereoisomers of adenosine, 9-β-l-(+)-adenosine, has been found to determine the increase of dry matter and other growth parameters, as it activates a rapid cascade of metabolic events throughout the plant [87]. This finding could explain the elevated dry matter data of HS plants, in comparison to WS and controls (Figure 1).

HS application also determined the activation of the terpenoid biosynthetic pathway, as highlighted by the increase of three saponins and two megastigmane sesquiterpenoids. Terpenoids are known to present antioxidant activity and to contribute to the stabilization of lipidic membranes [88].

Regarding the elemental content, we observed an increase of K, which is essential to the performance of multiple plant enzyme functions and is involved in plant heat tolerance strategies, regulating the metabolite pattern of higher plants. During abiotic stress conditions, K can act as a ROS detoxifying agent, improve CO_2_ fixation, and upregulate the activity of antioxidant enzymes; when high temperatures occur, K may work as an osmolyte and help to maintain stomatal conductance to prevent plant damages [89]. Differently from what was observed for WS plants, increased levels of K could indicate the development of a heat resistance strategy by RolD plants toward HS, in comparison with the other genetic modifications previously investigated. Our previous studies regarding *rolC*- and GR-modified *Nicotiana langsdorffii* exposed to HS, indeed, always showed a decrease of K (especially in the roots) and, in general, of all the main elements [10,21]. Moreover, it has been shown that an increased level of K has a positive impact on the energy status of plants, favoring ATP biosynthesis [65], in agreement with our finding of increased adenosine concentration.

## 3. Materials and Methods

### 3.1. Sample Preparation

#### 3.1.1. Plant Growth and Genetic Modification

Specimens of *Nicotiana langsdorffii Weinmann* were cultivated in vitro in the Laboratory of Plant Genetics, Department of Evolutionary Biology of the University of Florence. Details about cultivation have already been reported [15].

Wild-type (WT) plants were genetically modified by insertion of the *rolD* gene following the protocol of Horsch, 1988 [90]. Leaf disk transformation experiments were performed using *Agrobacterium tumefaciens* strain GV3101 and the binary vector pBin19::rolD. The procedure employed for plant genetic modification has been extensively described elsewhere [12,16,20]. The expression of the transgene was screened as previously described [20].

#### 3.1.2. Stress Inductions

Plants were exposed to heat stress (HS) and water stress (WS), as described by Scalabrin et al. [15]. The best temperature for the heat treatment was selected from preliminary studies conducted on *Nicotiana langsdorffii* WT plants, as previously described [14]. Three different types of samples were analyzed: non-stressed transgenic plants (RolD), HS plants (RolDHS), and WS plants (RolDWS).

#### 3.1.3. Plant Material Preparation

In order to remove the growth medium residues, plants were cleaned quickly with distilled water. After being frozen in liquid nitrogen, samples were freeze-dried in an Edward freeze-drying machine. Grinding and homogenizing were performed by a ball mill (MM 400, Retsch, Verder Scientific, Haan, Germany), working for 5 min with a vibration frequency of 20 Hz, to obtain a final fineness of ≈5 μm.

### 3.2. Metabolomics Analysis

#### 3.2.1. Experimental Procedure and Quality Control

The sample treatment procedure was based on the protocol of De Vos et al. [15], which has already been described in a previous study [15]. Briefly, the lyophilized and milled plant material (50 ± 0.5 mg) was weighed and the internal standard, phenyl-^13^C_6_ salicylic acid, was added. Samples were then extracted for 30 min by ultrasonic bath with 1.5 mL of MeOH:H_2_O 75:25 (*v*:*v*) acidified with formic acid 0.1%. After centrifugation (20 min at 14,000 rpm), the supernatant was collected, filtered with PTFE syringe filters (Ø 25 mm, 0.45 µm), and analyzed. Each sample was analyzed in three replicates to check the repeatability of the experiment. Blank samples were prepared and used to control contamination. The internal standard was used to check for errors during sample handling. Analyses were performed using an UltiMate 3000 (Dionex) coupled to an ESI-LTQ Orbitrap XL (Thermo Fisher Scientific, Waltham, MA, USA), as described by Scalabrin et al. [15]. The chromatographic analysis was performed by gradient elution with H_2_O and ACN acidified with 0.01% of formic acid on a SB-Aq Narrow Bore RR 2.1 × 150 mm, 3.5 µm column (Agilent Technologies, Wilmington, DE, USA). The ESI source was operated, in both negative and positive polarities, in full-scan modality at a resolving power of 60,000 (mass range 90–1500 *m*/*z*). Data-dependent acquisitions were also performed, obtaining a complete fragmentation pattern of the molecules.

#### 3.2.2. Data Treatment and Metabolite Identification

MetAlign [91,92] and MSClust [93] software were employed to perform mass spectra alignment, baseline correction, and mass signal clustering, as described by Scalabrin et al. [15]. Available online libraries (Metlin, HMDB, Dictionary of Natural Products, LIPID MAPS Structure Database) and literature were used to achieve metabolite identification, on the basis of the monoisotopic mass, the most probable molecular formula, and molecular fragmentation. The identification was carried out according to Sumner et al. [94].

### 3.3. Phytohormone Analysis

The method employed for the analysis of JA and SA has already been described elsewhere [14]. Briefly, 0.1 g of plant material was weighed and the internal standard, phenyl-^13^C_6_ salicylic acid, was added. The samples were extracted three times for 20 min with 1.5 mL of fresh MeOH acidified with HCl by centrifugation (14,000 rpm); the supernatants were collected, unified, and evaporated under a gentle stream of nitrogen until reaching a volume of 0.5 mL. Finally, 1 mL of water was added, and the extracts were filtrated with a syringe PTFE filter (Ø 25 mm, 0.45 mm) and analyzed. Three blank samples were also prepared, in order to check for possible contamination. Analyses were conducted using a UHPLC Dionex Ultimate3000 LC system coupled to a HESI-LTQ Orbitrap (Thermo Fisher Scientific, Waltham, MA, USA). The chromatographic separation was performed on a C18 phase 4 mm Synergy Hydro-RP 80 Å, 50 × 2 mm (Phenomenex^®^, Torrance, CA, USA) eluted with acetic acid 0.1% and MeOH. The ESI ion source was operated in negative ion mode, in full scan mode, at a resolving power of 30,000. The quantification was performed by means of an instrumental response factor containing the internal standard and the analytes at a known concentration, in order to reduce possible instrumental signal variations. Chromatograms were integrated with the LCquant 2.6.1 software (Thermo Fisher Scientific, Waltham, MA, USA).

### 3.4. Elemental Analysis

The sample treatment procedure followed the protocol already reported by Ranaldo et al. [23]. Aliquots of about 0.1 g of lyophilized samples were digested in 6 mL of ultrapure nitric acid (65%) and 4 mL of hydrogen peroxide (33%) using a temperature-controlled microwave oven (Ethos1-Milestone S.r.l. Advanced Microwave Digesting Labstation, Italy). For each batch of microwave digestion, two procedural blanks and eight samples were prepared. To assess the accuracy of the results, tomato leaf certified material NIST 1573a was used. Measurements were carried out using an ICP-LRMS (Agilent 7500 ORS—Agilent Technologies Inc. Santa Clara, CA, USA) equipped with an Octopole Reaction System (ORS) and an autosampler. The instrument was fitted out with a concentric nebulizer, a Peltier-cooled quartz spray chamber, and a quartz torch with a quartz injector tube. Optimization of instrumental performance, tuning details, and operating conditions have been previously reported [23]. Rhodium was used as an internal standard.

### 3.5. Statistical Analysis

Statistical differences among the samples were analyzed using Student’s t-test, by means of the Statistica 8.0 software (StatSoft, Inc., 2007, Tulsa, OK, USA). Differences were considered significant at a probability level of *p* < 0.01. Hierarchical cluster analysis (HCA) and heatmap were carried out using the Metaboanalyst 4.0 software [95].

## 4. Conclusions

This work studied the metabolomic responses of transgenic *N. langsdorffii* plants to abiotic stresses. A multi-approach analysis was conducted, with an examination of the content of two important phytohormones (jasmonic acid and salicylic acid) and tracing of the metabolomics and ionomics profiles, in order to obtain a complete evaluation of the plant metabolic status under abiotic stress conditions. The most relevant compounds for each kind of stress were statistically evaluated. Ninety-eight metabolites were identified, most of which belonged to the classes of lipids, acylsugars, glycosides, and amino acid derivatives. Stress application determined increases of the levels of phytohormones, indicating activation of the plant defense system. Changes in the levels of lipids, particularly lysolipids, acylsugars, and compounds related to the phenylpropanoid pathway, were also observed. WS determined the decrease of elements and of many compounds; amino acids and their derivatives were determined as key molecules in this kind of stress. HS induced a lower metabolic adjustment, in comparison to WS and to previous studies conducted on *rolC* and GR genetic modifications [14,15], indicating the minor impact of HS on *rolD* plants. Interestingly, RolDHS samples presented higher dry weight levels than the controls, as well as showing increased amounts of K and adenosine, suggesting the higher resistance of RolD plants to this kind of stress, in comparison to other genetic modifications. The results obtained in this study contribute to the elucidation of the mechanisms involved in the plant abiotic stress response and improves the knowledge about the potential of *rolD* gene insertion in resistance against water and heat stress. However, more investigations are needed, in order to highlight the possible effect of insertion of the *rolD* gene on the plant genome, to investigate whether the metabolic differences observed in this study were related to the expression of the *rolD* gene or to the knockdown gene mechanism.

## Figures and Tables

**Table 1:** Mean fresh weight (FW), mean dry weight (DW), and standard deviations (SD) of RolD: non-stressed transgenic plants; RolDWS: transgenic plants exposed to water stress; and RolDHS: transgenic plants exposed to heat stress samples (g).

	FW	DW
RolD	3.3	0.216 *
SD	1.2	0.08
RolDWS	3.4	0.34
SD	2.4	0.2
RolDHS	4.21	0.378 *
SD	0.6	0.06

* asterisks indicate statistically significant differences.

## Supplementary Materials

The following are available online at https://www.mdpi.com/2218-1989/10/8/310/s1, Table S1: Metabolites identified by untargeted metabolomics analysis at levels 2 and 3.

## References

[1] Bartwal A., Mall R., Lohani P., Guru S.K., Arora S. (2013). Role of Secondary Metabolites and Brassinosteroids in Plant Defense Against Environmental Stresses. J. Plant Growth Regul..

[2] Barnabás B., Jäger K., Fehér A. (2008). The effect of drought and heat stress on reproductive processes in cereals. Plant Cell Environ..

[3] Hasanuzzaman M., Nahar K., Fujita M., Vahdati K., Leslie C. (2009). Extreme Temperature Responses, Oxidative Stress and Antioxidant Defense in Plants. Abiotic Stress—Plant Responses and Applications in Agriculture.

[4] Larkindale J., Huang B. (2004). Changes of lipid composition and saturation level in leaves and roots for heat-stressed and heat-acclimated creeping bentgrass (*Agrostis stolonifera*). Environ. Exp. Bot..

[5] Yordanov I., Velikova V., Tsonev T. (2000). Plant responses to drought, acclimation and stress tolerance. Photosynthetica.

[6] Osmolovskaya N., Shumilina J., Kim A., Didio A., Grishina T., Bilova T., Keltsieva O.A., Zhukov V., Tikhonovich I., Tarakhovskaya E. (2018). Methodology of drought stress research: Experimental setup and physiological characterization. Int. J. Mol. Sci..

[7] Arbona V., Manzi M., De Ollas C., Gómez-Cadenas A. (2013). Metabolomics as a tool to investigate abiotic stress tolerance in plants. Int. J. Mol. Sci..

[8] Guo R., Shi L., Jiao Y., Li M., Zhong X., Gu F., Liu Q., Xia X., Li H. (2018). Metabolic responses to drought stress in the tissues of drought-tolerant and drought-sensitive wheat genotype seedlings. AoB Plants.

[9] Lipiec J., Doussan C., Nosalewicz A., Kondracka K. (2013). Effect of drought and heat stresses on plant growth and yield: A review. Int. Agrophysics.

[10] Malandrino M., Giacomino A., Karthik M., Zelano I., Fabbri D., Ginepro M., Fuoco R., Bogani P., Abollino O. (2017). Inorganic markers profiling in wild type and genetically modified plants subjected to abiotic stresses. Microchem. J..

[11] Wang W., Vinocur B., Altman A. (2003). Plant responses to drought, salinity and extreme temperatures: Towards genetic engineering for stress tolerance. Planta.

[12] Giannarelli S., Muscatello B., Bogani P., Spiriti M.M., Buiatti M., Fuoco R. (2010). Comparative determination of some phytohormones in wild-type and genetically modified plants by gas chromatography-mass spectrometry and high-performance liquid chromatography-tandem mass spectrometry. Anal. Biochem..

[13] Irdani T., Caroppo S., Ambrogioni L. (2003). Response of *Nicotiana tabacum* plants overexpressing a glucocorticoid receptor to *Meloidogyne incognita* (Nematoda Tylenchida) infestation. Redia.

[14] Scalabrin E., Radaelli M., Capodaglio G. (2016). Simultaneous determination of shikimic acid, salicylic acid and jasmonic acid in wild and transgenic Nicotiana langsdorffii plants exposed to abiotic stresses. Plant Physiol. Biochem..

[15] Scalabrin E., Radaelli M., Rizzato G., Bogani P., Buiatti M., Gambaro A., Capodaglio G. (2015). Metabolomic analysis of wild and transgenic *Nicotiana langsdorffii* plants exposed to abiotic stresses: Unraveling metabolic responses. Anal. Bioanal. Chem..

[16] Bettini P., Michelotti S., Bindi D., Giannini R., Capuana M., Buiatti M. (2003). Pleiotropic effect of the insertion of the *Agrobacterium rhizogenes rolD* gene in tomato (*Lycopersicon esculentum* Mill.). Theor. Appl. Genet..

[17] Palazón J., Cusidó R.M., Roig C., Piñol M.T. (1998). Expression of the rol C gene and nicotine production in transgenic roots and their regenerated plants. Plant Cell Rep..

[18] Del Bubba M., Ancillotti C., Checchini L., Ciofi L., Fibbi D., Gonnelli C., Mosti S. (2013). Chromium accumulation and changes in plant growth, selected phenolics and sugars of wild type and genetically modified *Nicotiana langsdorffii*. J. Hazard. Mater..

[19] Mauro M.L., Costantino P., Bettini P.P. (2017). The never ending story of rol genes: A century after. Plant Cell. Tissue Organ Cult..

[20] Fuoco R., Bogani P., Capodaglio G., Del Bubba M., Abollino O., Giannarelli S., Spiriti M.M., Muscatello B., Doumett S., Turetta C. (2013). Response to metal stress of *Nicotiana langsdorffii* plants wild-type and transgenic for the rat glucocorticoid receptor gene. J. Plant Physiol..

[21] Ardini F., Soggia F., Abelmoschi M.L., Magi E., Grotti M. (2016). Effect of heat stress on the ionomic profile of *Nicotiana langsdorffii* wild-type and mutant genotypes. Int. J. Environ. Anal. Chem..

[22] Ancillotti C., Bogani P., Biricolti S., Calistri E., Checchini L., Ciofi L., Gonnelli C., Del Bubba M. (2015). Changes in polyphenol and sugar concentrations in wild type and genetically modified *Nicotiana langsdorffii* Weinmann in response to water and heat stress. Plant Physiol. Biochem..

[23] Ranaldo M., Toscano G., Radaelli M., Scalabrin E., Capodaglio G. (2015). *Nicotiana langsdorffii* wild type and genetically modified exposed to chemical and physical stress: Changes in element content. Int. J. Environ. Anal. Chem..

[24] Trovato M., Maras B., Linhares F., Costantino P. (2001). The plant oncogene *rolD* encodes a functional ornithine cyclodeaminase. Proc. Natl. Acad. Sci. USA.

[25] Michaletti A., Naghavi M.R., Toorchi M., Zolla L., Rinalducci S. (2018). Metabolomics and proteomics reveal drought-stress responses of leaf tissues from spring-wheat. Sci. Rep..

[26] Dastogeer K.M.G., Li H., Sivasithamparam K., Jones M.G.K., Du X., Ren Y., Wylie S.J. (2017). Metabolic responses of endophytic *Nicotiana benthamiana* plants experiencing water stress. Environ. Exp. Bot..

[27] Yamada M., Morishita H., Urano K., Shiozaki N., Yamaguchi-Shinozaki K., Shinozaki K., Yoshiba Y. (2005). Effects of free proline accumulation in petunias under drought stress. J. Exp. Bot..

[28] Kumar D. (2014). Salicylic acid signaling in disease resistance. Plant Sci..

[29] Guy C., Kaplan F., Kopka J., Selbig J., Hincha D.K. (2008). Metabolomics of temperature stress. Physiol. Plant..

[30] Larkindale J., Hall J.D., Knight M.R., Vierling E., Larkindale J., Hall J.D., Knight M.R., Vierling E. (2005). Heat Stress Phenotypes of Arabidopsis Mutants Implicate Multiple Signaling Pathways in the Acquisition of Thermotolerance Published by: American Society of Plant Biologists (ASPB) Stable URL: https://www.jstor.org/stable/4629891 REFERENCES Linked refe. Plant Physiol..

[31] Khan M.I.R., Fatma M., Per T.S., Anjum N.A., Khan N.A. (2015). Salicylic acid-induced abiotic stress tolerance and underlying mechanisms in plants. Front. Plant Sci..

[32] Kazan K. (2015). Diverse roles of jasmonates and ethylene in abiotic stress tolerance. Trends Plant Sci..

[33] Clarke S.M., Cristescu S.M., Miersch O., Harren F.J.M., Wasternack C., Mur L.A. (2009). Jasmonates act with salicylic acid to confer basal thermotolerance in *Arabidopsis thaliana*. New Phytol..

[34] Wasternack C. (2007). Jasmonates: An Update on Biosynthesis, Signal Transduction and Action in Plant Stress Response, Growth and Development. Ann. Bot..

[35] Upchurch R.G. (2008). Fatty acid unsaturation, mobilization, and regulation in the response of plants to stress. Biotechnol. Lett..

[36] Murakami Y. (2000). Trienoic Fatty Acids and Plant Tolerance of High Temperature. Science.

[37] Chen J., Li M., Yang R., Luo Q., Xu J., Ye Y., Yan X. (2016). Profiling lipidome changes of *Pyropia haitanensis* in short-term response to high-temperature stress. J. Appl. Phycol..

[38] Cowan A.K. (2006). Phospholipids as plant growth regulators. Plant Growth Regul..

[39] Ryu S.B., Karlsson B.H., Ozgen M., Palta J.P. (1997). Inhibition of phospholipase D by lysophosphatidylethanolamine, a lipid-derived senescence retardant. Proc. Natl. Acad. Sci. USA.

[40] Hu L., Bi A., Hu Z., Amombo E., Li H., Fu J. (2018). Antioxidant metabolism, photosystem ii, and fatty acid composition of two tall fescue genotypes with different heat tolerance under high temperature stress. Front. Plant Sci..

[41] Tarazona P., Feussner K., Feussner I. (2015). An enhanced plant lipidomics method based on multiplexed liquid chromatography-mass spectrometry reveals additional insights into cold- and drought-induced membrane remodeling. Plant J..

[42] Joshi V., Joung J.G., Fei Z., Jander G. (2010). Interdependence of threonine, methionine and isoleucine metabolism in plants: Accumulation and transcriptional regulation under abiotic stress. Amino Acids.

[43] Dharmawardhana P., Ren L., Amarasinghe V., Monaco M., Thomason J., Ravenscroft D., McCouch S., Ware D., Jaiswal P. (2013). A genome scale metabolic network for rice and accompanying analysis of tryptophan, auxin and serotonin biosynthesis regulation under biotic stress. Rice.

[44] Bielach A., Hrtyan M., Tognetti V.B. (2017). Plants under Stress: Involvement of Auxin and Cytokinin. Int. J. Mol. Sci..

[45] Wang C., Yang A., Yin H., Zhang J. (2008). Influence of Water Stress on Endogenous Hormone Contents and Cell Damage of Maize Seedlings. J. Integr. Plant Biol..

[46] Das A., Rushton P., Rohila J. (2017). Metabolomic Profiling of Soybeans (*Glycine max* L.) Reveals the Importance of Sugar and Nitrogen Metabolism under Drought and Heat Stress. Plants.

[47] Good G.A., Zaplachinski S.T. (1994). The effects of drought stress on free amino acid accumulation and protein synthesis in Brassica napus. Physiol. Plant..

[48] Moradi P., Ford-Lloyd B., Pritchard J. (2017). Metabolomic approach reveals the biochemical mechanisms underlying drought stress tolerance in thyme. Anal. Biochem..

[49] Alcázar R., Altabella T., Marco F., Bortolotti C., Reymond M., Koncz C., Carrasco P., Tiburcio A.F. (2010). Polyamines: Molecules with regulatory functions in plant abiotic stress tolerance. Planta.

[50] Vincent D., Lapierre C., Pollet B., Cornic G., Negroni L., Zivy M. (2005). Water Deficits Affect Caffeate O-Methyltransferase, Lignification, and Related Enzymes in Maize Leaves. A Proteomic Investigation. Plant Physiol..

[51] Bassard J.E., Ullmann P., Bernier F., Werck-Reichhart D. (2010). Phenolamides: Bridging polyamines to the phenolic metabolism. Phytochemistry.

[52] Verma S., Mishra S.N. (2005). Putrescine alleviation of growth in salt stressed *Brassica juncea* by inducing antioxidative defense system. J. Plant Physiol..

[53] Macoy D.M., Kim W.Y., Lee S.Y., Kim M.G. (2015). Biosynthesis, physiology, and functions of hydroxycinnamic acid amides in plants. Plant Biotechnol. Rep..

[54] Kim J., Kang K., Gonzales-vigil E., Shi F., Jones A.D., Barry C.S., Last R.L. (2012). Striking natural diversity in glandular trichome acylsugar composition is shaped by variation at the acyltransferase2 Locus in the wild tomato Solanum habrochaites. Plant Physiol..

[55] Glas J.J., Schimmel B.C.J., Alba J.M., Escobar-bravo R., Schuurink R.C., Kant M. (2012). Plant Glandular Trichomes as Targets for Breeding or Engineering of Resistance to Herbivores. Int. J. Mol. Sci..

[56] Sun L.X., Fu W.W., Li W., Bi K.S., Wang M.W. (2006). Diosgenin glucuronides from Solanum lyratum and their cytotoxicity against tumor cell lines. Z. Naturforsch. Sect. C J. Biosci..

[57] Kumar P.M., Murugan K., Kovendan K., Subramaniam J., Amaresan D. (2012). Mosquito larvicidal and pupicidal efficacy of *Solanum xanthocarpum* (Family: Solanaceae) leaf extract and bacterial insecticide, *Bacillus thuringiensis*, against *Culex quinquefasciatus* Say (Diptera: Culicidae). Parasitol. Res..

[58] El-Sayed A.A., Razin A.M., Swaefy H.M.F., Mohamed S.M., Abou-Aitah K.E.A. (2008). Effect of Water Stress on Yield and Bioactive Chemical Constituents of Tribulus Species. J. Appl. Sci. Res..

[59] Narula A., Kumar S., Srivastava P.S. (2005). Abiotic metal stress enhances diosgenin yield in *Dioscorea bulbifera* L. cultures. Plant Cell Rep..

[60] Parent G.J., Giguère I., Mageroy M., Bohlmann J., MacKay J.J. (2018). Evolution of the biosynthesis of two hydroxyacetophenones in plants. Plant Cell Environ..

[61] Miguel M.G., Barroso J.G. (1994). Accumulation of stress metabolites in cell suspension cultures of *Hyoscyamus albus*. Phytochemistry.

[62] Chong J., Pierrel M.-A., Atanassova R., Werck-Reichart D., Fritig B., Saindrenan P. (2001). Free and Conjugated Benzoic Acid in Tobacco Plants and Cell Cultures. Induced Accumulation upon Elicitation of Defense Responses and Role as Salicylic Acid Precursors. Plant Physiol..

[63] Väisänen E.E., Smeds A.I., Fagerstedt K.V., Teeri T.H., Willför S.M., Kärkönen A. (2015). Coniferyl alcohol hinders the growth of tobacco BY-2 cells and *Nicotiana benthamiana* seedlings. Planta.

[64] Widhalm J.R., Dudareva N. (2015). A familiar ring to it: Biosynthesis of plant benzoic acids. Mol. Plant.

[65] Barker A.V., Bloem E., Brown P.H., Bryson G.M., Datnoff L.E., De Kok L.J., Drihem K., Dunn M.A., Gorham J., Graham R.D., Barker A.V., Pilbeam J.D. (2007). Handbook of Plant Nutrition.

[66] Meisch H., Becker L.J.M. (1981). Vanadium in photosynthesis of *Chlorella fusca* and higher plants. Biochim. Biophys. Acta.

[67] Pokotylo I., Kravets V., Martinec J., Ruelland E. (2018). The phosphatidic acid paradox: Too many actions for one molecule class? Lessons from plants. Prog. Lipid Res..

[68] Djanaguiraman M., Prasad P.V.V., Kumari J., Rengel Z. (2019). Root length and root lipid composition contribute to drought tolerance of winter and spring wheat. Plant Soil.

[69] Browse J., Warwick N., Somerville C.R., Slack C.R. (1986). Fluxes through the prokaryotic and eukaryotic pathways of lipid synthesis in the ‘16:3′ plant *Arabidopsis thaliana*. Biochem. J..

[70] Higashi Y., Saito K. (2019). Lipidomic studies of membrane glycerolipids in plant leaves under heat stress. Prog. Lipid Res..

[71] Simon-Sarkadi L., Kocsy G., Várhegyi Á., Galiba G., De Ronde J.A. (2006). Stress-induced changes in the free amino acid composition in transgenic soybean plants having increased proline content. Biol. Plant..

[72] Zeier J. (2013). New insights into the regulation of plant immunity by amino acid metabolic pathways. Plant Cell Environ..

[73] Staswick P.E., Serban B., Rowe M., Tiryaki I., Maldonado M.T., Maldonado M.C., Suza W. (2005). Characterization of an Arabidopsis Enzyme Family That Conjugates Amino Acids to Indole-3-Acetic Acid. Plant Cell.

[74] Gianfagna T.J., Davies P.J. (1983). The transport of substances out of developing fruits in relation to the induction of apical senescence in *Pisum sativum* line G2. Physiol. Plant..

[75] Staswick P.E., Tiryaki I. (2004). The Oxylipin Signal Jasmonic Acid Is Activated by an Enzyme That Conjugates It to Isoleucine in Arabidopsis. Plant Cell.

[76] Torras-Claveria L., Jáuregui O., Codina C., Tiburcio A.F., Bastida J., Viladomat F. (2012). Analysis of phenolic compounds by high-performance liquid chromatography coupled to electrospray ionization tandem mass spectrometry in senescent and water-stressed tobacco. Plant Sci..

[77] Chen Z., Watanabe T., Shinano T., Ezawa T., Wasaki J., Kimura K., Osaki M., Zhu Y.G. (2009). Element interconnections in Lotus japonicus: A systematic study of the effects of element additions on different natural variants. Soil Sci. Plant Nutr..

[78] Ahanger M.A., Agarwal R.M. (2017). Potassium up-regulates antioxidant metabolism and alleviates growth inhibition under water and osmotic stress in wheat (*Triticum aestivum* L). Protoplasma.

[79] Kant S., Kafkafi U. (2002). Potassium and Abiotic Stresses in Plants. Potassium for Sustainable Crop Production.

[80] Ahanger M.A., Morad-Talab N., Abd-Allah E.F., Ahmad P., Hajiboland R. (2016). Plant growth under drought stress: Significance of mineral nutrients. Water Stress Crop Plants A Sustain. Approach.

[81] Sun G., Strebl M., Merz M., Blamberg R., Huang F.C., McGraphery K., Hoffmann T., Schwab W. (2019). Glucosylation of the phytoalexin N-feruloyl tyramine modulates the levels of pathogen-responsive metabolites in *Nicotiana benthamiana*. Plant J..

[82] Han F., Chen H., Li X.J., Yang M.F., Liu G.S., Shen S.H. (2009). A comparative proteomic analysis of rice seedlings under various high-temperature stresses. Biochim. Biophys. Acta Proteins Proteom..

[83] Marti G., Erb M., Boccard J., Glauser G., Doyen G.R., Villard N., Robert C.A.M., Turlings T.C.J., Rudaz S., Wolfender J.L. (2013). Metabolomics reveals herbivore-induced metabolites of resistance and susceptibility in maize leaves and roots. Plant. Cell Environ..

[84] Narayanan S., Prasad P.V.V., Welti R. (2016). Wheat leaf lipids during heat stress: II. Lipids experiencing coordinated metabolism are detected by analysis of lipid co-occurrence. Plant Cell Environ..

[85] Otsuru M., Yu Y., Mizoi J., Kawamoto-fujioka M., Wang J., Fujiki Y., Nishida I. (2013). Mitochondrial Phosphatidylethanolamine Level Modulates Cyt c Oxidase Activity to Maintain Respiration Capacity in Arabidopsis thaliana Rosette Leaves. Plant Cell Physiol..

[86] Naeem M., Khan A., Masroor M. (2012). Moinuddin Triacontanol: A potent plant growth regulator in agriculture. J. Plant Interact..

[87] Waqas M., Shahzad R., Khan A.L., Asaf S., Kim Y.H., Kang S.M., Bilal S., Hamayun M., Lee I.J. (2016). Salvaging effect of triacontanol on plant growth, thermotolerance, macro-nutrient content, amino acid concentration and modulation of defense hormonal levels under heat stress. Plant Physiol. Biochem..

[88] Rodziewicz P., Swarcewicz B., Chmielewska K., Wojakowska A., Stobiecki M. (2014). Influence of abiotic stresses on plant proteome and metabolome changes. Acta Physiol. Plant..

[89] Hasanuzzaman M., Bhuyan M.H.M.B., Nahar K., Hossain M.S., Al Mahmud J., Hossen M.S., Masud A.A.C., Moumita, Fujita M. (2018). Potassium: A vital regulator of plant responses and tolerance to abiotic stresses. Agronomy.

[90] Horsch R.B., Fry J., Hoffmann N., Neidermeyer J., Rogers S.G., Fraley R.T. (1988). Leaf disc transformation. Plant Mol. Biol. Man..

[91] Lommen A. (2009). MetAlign: Interface-driven, versatile metabolomics tool for hyphenated full-scan mass spectrometry data preprocessing. Anal. Chem..

[92] Lommen A., Van der Kamp H.J., Kools H.J., Van der Lee M.K., Van der Weg G., Mol H.G.J. (2012). metAlignID: A high-throughput software tool set for automated detection of trace level contaminants in comprehensive LECO two-dimensional gas chromatography time-of-flight mass spectrometry data. J. Chromatogr. A.

[93] Tikunov Y.M., Laptenok S., Hall R.D., Bovy A., De Vos R.C.H. (2012). MSClust: A tool for unsupervised mass spectra extraction of chromatography-mass spectrometry ion-wise aligned data. Metabolomics.

[94] Sumner L.W., Amberg A., Barrett D., Beale M.H., Beger R., Daykin C.A., Fan T.W.M., Fiehn O., Goodacre R., Griffin J.L. (2007). Proposed minimum reporting standards for chemical analysis. Metabolomics.

[95] Chong J., Soufan O., Li C., Caraus I., Li S., Bourque G., Wishart D.S., Xia J. (2018). MetaboAnalyst 4.0: Towards more transparent and integrative metabolomics analysis. Nucleic Acids Res..

