# Supplementary material for: Effects of Water Deficit and Heat Stress on Nicotiana langsdorffii Metabolomic Pattern Modified by Insertion of rolD Gene from Agrobacterium rhizogenes
Source: Metabolites. 2020 Jul 29;10(8):310. doi: 10.3390/metabo10080310 (PMC7463493; doi:10.3390/metabo10080310)
Supplement: Supplementary file 1 [file metabolites-10-00310-s001.pdf]

Effects of water deficit and heat stress on *Nicotiana Langsdorffii* metabolomic pattern modified by insertion of *rolD* gene from *Agrobacterium rhizogenes*.

Elisa Scalabrin[1*], Marta Radaelli[1], Gabriele Capodaglio[1]

[1] Department of Environmental Sciences, Informatics and Statistics, Ca'Foscari University of Venice, Via Torino 155, 30173 Mestre (Venezia), Italy; elisa.scalabrin@unive.it, marta.radaelli@unive.it, capoda@unive.it

* Correspondence: elisa.scalabrin@unive.it

Table S1: Metabolites identified by untargeted metabolomic analysis at levels 2 and 3. Diagnostic fragments are in bold.

Retention time (min)	Mass	Formula	Name	Fragments	Error (ppm)	ID Level
16,43	468.21310 (M - H)	$C_{25}H_{31}N_3O_6$	Dicaffeoyl spermidine	332.16077, 306.18170, 289.15533, 135.04524, 264.17065	-1,9	2
22,09	783,32617 (M - H)	$C_{34}H_{56}O_{20}$	Menthenetriol glucoside + ketoglutaric acid + octanoic acid	739.33136, 652.68506, 491.28287, 287.14825, 215.12827, 143.10721	-3,1	2
14,95	250,07155 (M - H)	$C_{12}H_{13}NO_5$	Phenylacetylaspartic acid	132.03020, 232.06131	-2,18	2
9,88	315,07129 (M - H)	$C_{13}H_{16}O_9$	Glucopyranosyloxyhydroxybenzoic acid	153.01921, 109.02940, 165.01924, 225.03976	-2,36	2
14,39	373.11310 (M - H)	$C_{16}H_{22}O_{10}$	Secologanate	358.08963, **211.06085**, 196.03746, 179.07130, 153.0557, 304.91241, 265.07080, 195.02966, 180.04237, 139.04002	-3,1	2
9,65	367.12387 (M - H)	$C_{14}H_{24}O_{11}$	Acetyl methyl glucuronic acid + gamma hydroxyvaleric acid	349.11319, 323.09775, 249.06126, 231.05061	-3,5	2

15,06	472,2439 (M-H)	$C_{23}H_{40}NO_7P$	LysoPE (18:4; 0:0)	381.17514, 350.20694, 308.19662, 186.16068, 121.02934, 137.06053, 266.08671	-5,32	2
13,23	372.12909 (M + FA - H) 326.12372 (M - H)	$C_{15}H_{21}NO_7$	Phenylalanine glucopyranose	304.91229, 236.09235, 179.05600, 161.04530	-2,4	2
13,03	381,13959	$C_{15}H_{26}O_{11}$	Acetylmethyl glucuronic acid-hydroxy methyl pentanoic acid	**249.06136, 231.05087**, 206.06825	-2,4	2
16,09	385,11325	$C_{17}H_{22}O_{10}$	Acetyl methyl glucuronic acid hydroxy acetophenone	267.07184, **249.06114**	-2,3	2
11,20	399,13171	$C_{15}H_{28}O_{10}S$	Propyl -glucopyranosyl-2-tio-glucopyranoside	136,99147	-3,3	2
33,37	433,23569	$C_{21}H_{39}O_7P$	PA (18:2/0:0)	152.99603, 171.00659	-0,5	2
10,70	395,02789	$C_{13}H_{16}O_{12}S$	Periodic leaf movement factor, PLMF 3 (Glucopyranosyloxyhydroxybenzoic acid sulfate)	351.03842, 377.01718, 241.00220, 153.01930, 241.00194, 253.02708, 109.02946	-3,3	2
14,05	504.17111 (M + FA - H) 458.16510 (M - H)	$C_{20}H_{29}NO_{11}$	Cinnamide diglucoside	357.10263, 311.09732, 293.08664, 251.07672, 221.06549, 191.05542, 161.04521	-3,6	2
18,42	461.23703 (M + FA - H) 415.23175 (M - H)	$C_{21}H_{36}O_8$	Sesquiterpenoid		-4,7	3
22,44	485,13596	$C_{24}H_{26}N_2O_7S$	Caffeoyl-indole-3-acetyl methionine	467.12592, 441.14661, 423.13562, 349.08429, **305.09497**, 232.09683, 190.03238	-5,8	2

19,59	483.20779 (M + FA - H) 437.20227 (M - H)	$C_{19}H_{34}O_{11}$	Caprylic acid + xylopyranosyl glucopyranoside	293,08746	-4,04	2
26,24	552.24249 (M + FA - H) 506.23688 (M - H)	$C_{26}H_{37}NO_9$	Octahydro-quinolizine-Glucopyranosyl-hydroxy-methoxy-cinnamoyl	362.12244, 200.07079, 305.15894, 260.09140, 117.03468	-2,28	2
13,61	491.13986 (M + FA - H) 445.13428 (M - H)	$C_{19}H_{26}O_{12}$	Benzoic acid difructofuranose dianydrhyde	323.09787, 305.08676, 263.07663, 221.06628, 179.05598	-3	2
19,94	555,22815	$C_{29}H_{40}O_{15}$	Menthenetriol diglucoside	509.22238, 491.21146, 431.19080, 389.18036, 365.10730, **305.15964, 347.17001**, 233.13889, 161.04529, 179.05534, 143.03474	-2,3	2
22,06	649.30432 (M + 2FA - 2H) 1207.60474 (M - H)	$C_{58}H_{96}O_{26}$	Diosgenin + 3glucoses + 1 dehydroxyglucose + 1 methyldehydroxyglucose (Methyldichotomin)	626.30121 (-46), 603.29865 (-46), 1061.54749 (-146)	-5,8	2
18,04	645.25842 (M + FA - H) 599.25323 (M - H)	$C_{25}H_{44}O_{16}$	Menthenetriol diglucoside + glycerol + galactose	581.24353, **305.15909**, 497.22095, 455.13800, **347.16897,** 275.07568, 233.13835, 215.12788, 161.04489, 179.05542	-4,7	2
26,62	653.26202 (M + FA - H) 607.25720 (M - H)	$C_{27}H_{44}O_{15}$	Acylcsucrose (S4:15)	627.54944, **565.24677**, 56077814, 495.20340, **481.18893**, 477.19397, 463.17798	-5,8	2

18,26	645.25812 (M +FA - H)<br>599.25323 (M - H)	$C_{25}H_{44}O_{16}$	Menthenetriol diglucoside + carboxyethylglucose	581.24353, 275.07599, 539.23181, 497.22095, 455.13800, 389.17941, 347.16898, 305.15884, 161.04489, 215.12823, 143.10721, 233.13835	-3,1	2
27,16	721.36536 (M + FA - H)<br>675.35974 (M - H)	$C_{33}H_{56}O_{14}$	Diglucopyranosyl linolenylglycerol (C18:3)	415.14557, 397.13477, 235.08237	0,2	2
33,01	723.38110 (M + FA - H)<br>677.37512 (M - H)	$C_{33}H_{57}O_{14}$	Acylsucrose (S3:21)	**593.31763**, **551.27081**, 533.26013	-0,38	2
35,08	751.44861 (M + FA - H)<br>705.44324 (M - H)	$C_{36}H_{66}O_{13}$	Dirhamnolipid C24:0 (C8:0, C16:0)	561.32794, 543.31775	0,2	2
35,58	765.42755 (M + FA - H)<br>719.42200 (M - H)	$C_{36}H_{64}O_{14}$	Acylsucrose (S3:24)	**593.31781**, **575.30658**, **467.21338**, 449.20279	-0,4	2
33,54	773.43237 (M +FA - H)<br>727.42731 (M - H)	$C_{38}H_{64}O_{13}$	Dirhamnolipid C25:3 (C18:3, C7:0)	583.31195, 565.30157, 277.21729	-0,144	2
34,42	775.44818 (M + FA - H)<br>729.44269 (M - H)	$C_{38}H_{66}O_{13}$	Dirhamnolipid C25:2 (C18:2, C7:0)	585.32806, 567.31610, 279.23318	-0,5	2
37,25	807.43829 (M + FA - H)<br>761.43182 (M - H)	$C_{38}H_{66}O_{15}$	Acylsucrose S4:26	719.42218, 635.32861, **593.31799**, **467.21503**, **575.30829**, 449.20309, 621.34656	-1,4	2
15,79	470.22781 (M - H)	$C_{23}H_{38}O_7NP$	LysoPE (18:4; 0:0)	334.17599, 291.17035, 121.02929, 137.06046, 169.13408, 180.06590, 237.12361, 249.12352	-7,3	3
19,01	579.20538 (M - H)	$C_{28}H_{36}O_{13}$	Syringaresinol O-beta-D-glucoside	417,15314	-5,06	2
21,50	569.26837 (M + 2FA - 2H)<br>1047.53406 (M-H)	$C_{51}H_{84}O_{22}$	Diosgenin + 3glucoses + 1 hydroxyglucose (methylprotodioscin)	546.26544 (-46) 523.26245 (-92)	-4,48	2

22,59	409.20578 (M + FA - H)<br>363.20068 (M - H)	$C_{17}H_{32}O_8$	Allylglucose + caprylic acid	220.31882, 143.10709	-4,8	2
21,02	642.29803 (M + 2FA - 2H)<br>1193.59351 (M - H)	$C_{57}H_{94}O_{26}$	Diosgenin + 3 glucoses + 2 dehydroxyglucose (Dichotomin)	619.29651 (-46), 596.29382 (-46), 523.26288 (-146), 1031.53821, 450.23441, 1047.53345, 901.47559, 1029.52332, 883.46600, **755.41864**, **737.40845**	-3,05	2
22,92	1175,58032	$C_{57}H_{92}O_{25}$	Diosgenin + 2 glucoses + 3 hydroxyglucoses	1029.52246 (-146), 883.46417 (-146), **737.40668** (-146), **557.34369** (-180), 865.45385, 806.02380, 766.13739, 719.39764, 671.37610, 653.36548, **575.35547**, 599.04858, 509.32275	-4,4	2
26,06	575.30457 (M+FA-H)<br>529.29895 (M-H)	$C_{28}H_{42}O_6N_4$	Bisdihydrocaffeoylspermine	511.28836, 407.19012, 293.21066, 253.09164, 275.20032	-6,7	2
20,23	553,21173	$C_{23}H_{38}O_{15}$	Menthenetriol diglucoside	509.22156, 389.17978, **347.16946**, 305.15915, 263.07602, 233.13853, 161.04503, 179.05545	-3,7	3
21,34	435,09119	$C_{20}H_{20}O_{11}$	Glucopyranosyl hydroxybenzoyl dihydroxybenzoic acid	**297.06003**, **315.07043,** 152.01093, 179.03415	-6,5	2
12,27	312.09442 (M + FA - H)<br>266.08936 (M - H)	$C_{10}H_{13}N_5O_4$	Adenosine	242.94350, 134.04739	-0,44	2
35,55	277,2171	$C_{18}H_{30}O_2$	Linolenic acid	259.20651, 233.22757	-0,7	2
22,43	312,12228	$C_{18}H_{19}NO_4$	Feruloyltyramine	297.09930, 270.11288, 242.94244, **178.05029,** 148.05272, **135.04482**	-5,9	2

12,72	372,09241	$C_{16}H_{23}NO_9$	Acetyl methyl glucuronic acid isonicotinic acid	**249.06108, 231.05063**	-3,2	2
36,65	279,23283	$C_{18}H_{32}O_2$	Linoleic acid	261,22223	-0,4	2
16,57	403.15964 (M + FA - H) 357.15430 (M - H)	$C_{17}H_{26}O_8$	Sesquiterpenoid	161,04518	-3,3	3
14,64	447.14957 (M + FA - H) 401.14401 (M - H)	$C_{15}H_{26}O_{10}$	Benzylalcohol hexose-pentose	269.10226, 293.08694, 356.13223, 233.06602, 161.04523, 277.09036, 251.09190, 262.06754	-3,2	2
14,11	451.21786 (M +FA -H) 405.21252 (M - H)	$C_{19}H_{34}O_9$	Megastigmenetetrol-glucopyranoside (Sesquiterpenoid)	340.12256, 243.16002, 225.14966, 179.05597, 167.10770, 149.09705, 143.03485	-1,1	2
28,01	474,26242	$C_{23}H_{42}NO_7P$	LysoPE (18:3/0:0)	277.21722, 233.22748, 259.20682	-0,4	2
26,30	623,23627	$C_{36}H_{36}N_2O_8$	Grossamide	486.15283, 460.17346, 445.14996, 297.11136, 283.09583, 268.07312, 471.12894, 427.13953, 352.08035	-5,8	2
32,28	651.41113 (M + FA - H) 605.40533 (M - H)	$C_{36}H_{58}O_8$	Saponin		-0,92	3
28,14	638,33118	$C_{29}H_{54}NO_{12}P$	LysoPE (18:2/0:0) + hexose	476.27805, 518.28864, 279.23279	-0,05	2
34,03	637,43188	$C_{36}H_{62}O_9$	Saponin		-0,35	3
42,19	673.46729 (M + FA)	$C_{38}H_{64}O_6$	Feruloyloxyfatty acid (C28:0)		-0,5	3

31,03	695.35022 (M + FA - H)<br>649.34393 (M - H)	$C_{31}H_{54}O_{14}$	Acylsucrose (S3:19)	**565.28619**, 551.27069, **523.23926**	-0,23	2
31,29	823.46887 (M - H)	$C_{44}H_{72}O_{12}S$	SQDG 35:6	805.46167, 585.24005, 567.22925, 415.14474, 397.13489, 661.41632 (-162), 643.40631 (-180), 187.09793	1,69	2
32,12	735.34393 (M + FA - H)	$C_{33}H_{54}O_{15}$	Acylsucrose (S4:21)		-0,223	2
32,75	737.36023 (M + FA - H)<br>691.35449 (M - H)	$C_{33}H_{56}O_{15}$	Acylsucrose (S4:21)	**649.34418,** 607.29761, **565.25488**, 523.932, **481.19174**	-0,223	2
33,47	751.37561 (M + FA - H)<br>705.37012 (M - H)	$C_{34}H_{58}O_{15}$	Acylsucrose (S4:22)	663.35950, **607.29712,** 555.28638, 537.25513, **481.19266**, 450.04047, 579.26575	-0,2	2
34,12	583.31543 (M - H)	$C_{27}H_{52}O_{11}S$	Deoxysulfoquinovosylmonoacyl glycerol	522.35614, **299.04395**, **225.00765**, 297.27969	-0,55	2
34,74	765.39105 (M + FA - H)<br>719.38678	$C_{35}H_{60}O_{15}$	Acylsucrose (S4:23)	677.37555, 635.32880, **593.28906**, **551.27112**, 566.47937, 548.46857	-0,48	2
35,53	779.40680 (M + FA - H)<br>733.40088 (M - H)	$C_{36}H_{62}O_{15}$	Tetraacylsucrose (S4:24)	**691.39069**, 607.29718, **565.28638**, **481.19226**, 439.18195	-0,9	2
41,51	837,48236	$C_{45}H_{74}O_{12}S$	SQDG 36:6 (C:18/C:18)	778.50342, 579.28290, 559.25781, **537.27264**, 481.71002, 413.66330	0,1	2
23,30	327,21603	$C_{18}H_{32}O_{5}$	Trihydroxyoctadecadienoic	**171.10214,** 211.13322, **229.14371**, 239.12776, 273.18454, 291.19528, 309.20554, 153.09113, 127.11237, 193.15909, 247.20549, 273.18460	-4,6	2

23,13	1113.52881 (M + FA - H) 1067.52161 (M - H)	$C_{50}H_{84}O_{24}$	Nicotianoside III	921.46710 (-146), 903.45825, 757.39960, 613.35699, **775.40753** (-146), 629.35046 (-146), 467.29852 (-146), **575.31903**, 595.34308	-4,4	2
28,87	476,27701	$C_{23}H_{44}NO_7P$	LysoPE (18:2/0:0)	279.23212, 261.22208	-2,6	2
25,71	609.26501 (M - H)	$C_{27}H_{47}O_{13}P$	PA (18:4;0:0) + glucose	563.26831, 467.21176, 447.21265 (-162), 429.20215 (-180), 293.21054, 315.04654, 275.20016, 241.01062, 235.16841, 265.21635, 244.75534	-5,15	2
14,05	172,09782	$C_8H_{15}NO_3$	Acetyl-leucine	130,08746	-0,56	2
7,14	203,08176	$C_{11}H_{12}N_2O_2$	Tryptophan	159.09236, 186.05562, 142.06607, 116.05032	-4,09	2
12,54	249,12405	$C_{13}H_{18}N_2O_3$	Caffeoylputrescine	207.11348, 135.04503	-1,6	2
11,56	192,0667	$C_{10}H_{11}NO_3$	Benzoylalanine	146.93811, 131.47552, 102.73841, 74.02512, 66.10155	0,43	2
11,92	215,13998	$C_{10}H_{20}N_2O_3$	Valyl valine	197.12953, 128.03546, 169.13451, 141.10333, 127.05141, 84.04576, 66.10163	-0,63	2
13,23	321,14499	$C_{16}H_{22}N_2O_5$	Acetyl-tyrosil valine	277,15555	-1,88	2
14,39	373,11261	$C_{16}H_{22}O_{10}$	Propyl gallate glucoside	358.08932, 304.91312, 328.13907, 211.06067, 195.02950, 153.05544, 180.04230, 179.07085, 196.03738	-3,77	2
13,77	343,09238	$C_{15}H_{20}O_9$	Dihydroxyacetophenone glucoside	328.08023, 299.10422, 275.07788, 181.05101, 166.02739, 234.04906	-3	2

20,53	381.164492 (M +FA -H) 305.15997 (M - H)	$C_{14}H_{26}O_7$	Glucopyranosyloxyoctanone	207.05090, 143.10780 (-162)	-1,62	2
13,91	386.14432 (M - H + FA) 340.13922 (M - H)	$C_{16}H_{22}NO_7$	Acetyltyramine glucoside	178,08691	-2,8	2
13,23	329,08661	$C_{14}H_{18}O_9$	Trihydroxyacetophenone glucopyranoside	167.03474, 311.1601	-1,9	2
15,67	321,15408	$C_{14}H_{26}O_8$	Octanoic acid glucoside	311.83716, 303.14395, 159.10197, 141.09163	-2,6	2
14,82	192,06606	$C_{10}H_{11}NO_3$	Phenylacetylglycine	74,0251	-2,8	2
11,99	340,13943	$C_{16}H_{23}NO_7$	Acetyl-hydroxy phenethylamine glucopyranose	178.08711, 161.04544, 143.03485, 125.02441, 101.02449	-2,19	2
15,65	399.09238 (M + FA - H) 353.08685 (M - H)	$C_{16}H_{18}O_9$	3CQA	191,03455	-2,7	1
15,87	461.16623 (M + FA - H) 415.16101 (M - H)	$C_{19}H_{28}O_{10}$	Phenylethanol apiofuranosyl glucopyranoside	311.09796, 293.08740, 191.05603, 161.04555, 149.04562, 131.03503, 113.02453	-4	2
32,28	481,25677	$C_{22}H_{43}O_9P$	PG (16:1/0:0)	253.21730, 245.04327, 152.99620	-0.8	2
32,09	431,22028	$C_{21}H_{37}O_7P$	PA(18:3/0:0)	152.99603, 363.21521, 171.00633	-0,309	2
19,32	483.20621 (M + FA - H) 437.20065 (M - H)	$C_{19}H_{34}O_{11}$	Octanoic acid + dyhydroxymethylene butanoyl beta glucopyranoside	403.69440, 293.08636, 191.05574, 149.04539, 131.03494, 125.02441, 101.02441, 89.02454	-4,99	2
18,66	513.21667 (M - H + FA) 467.21097 (M - H)	$C_{20}H_{36}O_{12}$	Sucrose octanoate	323.09671, 305.08640	-5,2	2

18,04	675.25775 (M + FA - H) 599.25262 (M - H)	$C_{25}H_{43}O_{16}$	Octenediol - diglucose - xylose	305.15884, 581.24194, 539.23181, 497.22095, 455.13800, 389.17941, 347.16898, 275.07568, 233.13835, 161.04489	-4,05	2
30,08	577,26654	$C_{27}H_{46}O_{11}S$	SQMGC18:3	299.04263, 225.00653, 277.21597	-3,925	2
17,65	245,09212	$C_{13}H_{14}N_2O_3$	Acetyl tryptophan	203.08199, 186.05530, 159.09239, 142.06580, 116.05039	-4,2	2
22,81	597.23712 (M + FA - H) 551.23236	$C_{25}H_{42}O_{16}$	Triacylsucrose (S3:12)	536.29572, **509.22107**, **467.17386**, 311.22119, 425.16483, 407.15314	-4,8	2
22,97	597.23712 (M + FA - H) 551.23236	$C_{25}H_{42}O_{16}$	Triacylsucrose (S3:12)	536.29572, **509.22107**, **467.17386**, 311.22119, 425.16483, 407.15314	-4,8	2
34,28	409,23572	$C_{19}H_{39}O_7P$	LPA(0:0/16:0)	152.99606, 369.90182, 340.20300, 171.00641	-0,107	2
27,92	653.30243 (M + AF - H) 607.29736 (M-H)	$C_{28}H_{48}O_{14}$	Triacylsucrose (S3:16)	523.23962, **509.22409**, 491.21301, 425.16415, 407.15527, 319.07587, 439.18204, 396.82724, 323.09824	-0,089	2
15,35	321,15442	$C_{14}H_{26}O_8$	Octanoic acid glucoside	303.14401, 159.10231, 141.09180, 119.03476, 101.02428	-3,35	2
28,30	681.29718 (M + FA - H) 635.29163 (M - H)	$C_{30}H_{49}O_{17}$	Tetraacylsucrose (S4:17)	593.28168, **551.23468, 509.22233**, 471.75043, 399.14078, 491.21207, **467.31448**	-0,32	2

28,01	679.28137 (M + FA - H)<br>633.27594 (M - H)	$C_{30}H_{47}O_{17}$	Tetraacylsucrose (S4:17)	591.26581, **551.23230, 509.22476**, 415.14536, 397.13455	-0,74	2
28,72	681.29718 (M + AF - H)<br>635.29163 (M -H)	$C_{30}H_{50}O_{17}$ + CH_2O_2	Tetraacylsucrose (S4:17)	593.28168, 551.23468, 509.22263, 399.14078, 491.21207	-3	2
15,98	353,11301	$C_{13}H_{24}NO_8S$	Homomethionine + quinic acid	309.12335, 191.05563	-5,5	2
30,42	709,32623	$C_{31}H_{51}O_{15}$ + CH_2O_2	Tetraacylsucrose (S4:19)	621.30927, 579.26245, 523.23724, 537.25281, 505.22699	-4.3	2
29,72	695.31165 (M + FA)	$C_{30}H_{50}O_{15}$ + CH_2O_2	Tetraacylsucrose (S4:18)	607.29517, 565.24805, 523.23773, 467.17517, 509.22223, 491.21164, 407.15445, 551.23254	-3.59	2
30,66	709,3277	$C_{31}H_{51}O_{15}$ + CH_2O_3	Tetraacylsucrose (S4:19)	621.30927, 523.23724, 537.25281, 565.25739, 421.17258	-1.6	2
27,33	721.36536 (M + AF - H)<br>675,35974 (M-H)	$C_{33}H_{56}O_{14}$	Digalactopyranosyl lineoylglicerol (C18:3)	397,13483	0,016	2
33,01	723,38092	$C_{33}H_{58}O_{14}$ + AF	Triacylsucrose (S3:21)	677.37500, 551.27081, 593.31763, 533.26019	0,33	2
22,47	453,13809	$C_{21}H_{26}O_{11}$	Dihydroferulic acid glucuronide + methylfurane	435.12799, 371.09631, 353.08560, 331.10107, 249.06006	-4,7	2
41,73	787,46674	$C_{41}H_{72}O_{12}S$	SQDG 16:3 18:0	537.27368, 559.25854	-0,5	2
23,76	1007,46606	$C_{47}H_{76}O_{23}$	Nicotianoside IV	963.47504, 903.45404, 921.46436, 775.40692, 757.39697	-4,36	2
26,98	1059.53882 (M + FA - H)<br>1013.53107 (M - H)	$C_{51}H_{82}O_{20}$	Diosgenin tetraglicoside	868.47736, 722.41968, 576.35822	-0,866	2

19,51	1074.54419 (M - H + FA) 1028.53674 (M - H)	$C_{51}H_{83}NO_{20}$	Solanidine tetraglycoside		-6,6	3
20,40	1119.63293 (M + FA - H)	$C_{51}H_{95}NO_{22}$	Glykoalkaloid		-7,8	3
17,37	675.26807 (M + FA - H) 629.26263 (M - H)	$C_{26}H_{45}O_{17}$	Sucrose + hexose octanoate	611.25220, 551.23083, 467.21072, 341.10675, 323.09659	-5,7	2
1,67	116.0709 (M + H)	$C_5H_9NO_2$	Proline		-2.58	2
1,54	175.1192 (M + H)	$C_6H_{14}N_4O_2$	Arginine	116.0706, 130.0961	1,71	2
1,7	132.1015 (M + H)	$C_6H_{13}NO_2$	Leucine +Isoleucine	86.0967	3,02	2
1,7	166.0857 (M + H)	$C_9H_{11}NO_2$	Phenylalanine	120.0804	3,34	2
1,65	182.0807 (M + H)	$C_9H_{11}NO_3$	Tyrosine	165.0548, 136.0750	2,19	2
2,05	174.0527 (M-H)	$C_{10}H_9NO_2$	3-Indole acetic acid	130.0639, 128.0485	1,14	2
14,28	265,14344 (M + H)	$C_{15}H_{20}O_4$	Abscisic acid	247.1290, 187.1115	3.449	2
